# Automating virtual dental implant planning: can artificial intelligence match clinical expertise?

**DOI:** 10.1093/dmfr/twag015

**Published:** 2026-04-22

**Authors:** Bahaaeldeen M Elgarba, Eslam Abdelwahab Dawood, Rocharles Cavalcante Fontenele, Pierre Lahoud, Jan Meeus, Reinhilde Jacobs

**Affiliations:** OMFS-IMPATH Research Group, Department of Imaging and Pathology, Faculty of Medicine, KU Leuven, Leuven 3000, Belgium; Department of Prosthodontics, Faculty of Dentistry, Tanta University, Tanta 31511, Egypt; Department of Oral and Maxillofacial Surgery, University Hospitals Leuven, Leuven 3000, Belgium; OMFS-IMPATH Research Group, Department of Imaging and Pathology, Faculty of Medicine, KU Leuven, Leuven 3000, Belgium; Department of Prosthodontics, Faculty of Dentistry, Tanta University, Tanta 31511, Egypt; Department of Oral and Maxillofacial Surgery, University Hospitals Leuven, Leuven 3000, Belgium; OMFS-IMPATH Research Group, Department of Imaging and Pathology, Faculty of Medicine, KU Leuven, Leuven 3000, Belgium; Department of Oral and Maxillofacial Surgery, University Hospitals Leuven, Leuven 3000, Belgium; Department of Stomatology, Public Health and Forensic Dentistry, Division of Oral Radiology, School of Dentistry of Ribeirão Preto, University of São Paulo (USP), Ribeirão Preto 14040-904, Brazil; OMFS-IMPATH Research Group, Department of Imaging and Pathology, Faculty of Medicine, KU Leuven, Leuven 3000, Belgium; Department of Oral and Maxillofacial Surgery, University Hospitals Leuven, Leuven 3000, Belgium; Division of Periodontology and Oral Microbiology, Department of Oral Health Sciences, KU Leuven, Leuven 3000, Belgium; Department of Conservative Dentistry, Periodontology and Digital Dentistry, LMU University Hospital, LMU Munich, Munich D-80539, Germany; Department of Oral and Maxillofacial Surgery, University Hospitals Leuven, Leuven 3000, Belgium; OMFS-IMPATH Research Group, Department of Imaging and Pathology, Faculty of Medicine, KU Leuven, Leuven 3000, Belgium; Department of Oral and Maxillofacial Surgery, University Hospitals Leuven, Leuven 3000, Belgium; Department of Dental Medicine, Karolinska Institute, Stockholm 171 77, Sweden

**Keywords:** artificial intelligence, dental implant, implant dentistry, 3D imaging, cone-beam CT

## Abstract

**Objectives:**

This study evaluated a validated artificial intelligence (AI) tool for virtual implant planning by comparing its performance with expert-planned and clinically placed implants in single-tooth rehabilitation cases.

**Methods:**

Pre- and postoperative cone-beam CT (CBCT) scans were retrospectively collected from 32 single-tooth implant cases. Actual implants were placed by expert clinicians using a human intelligence (HI)-based approach guided by the preoperative CBCT. The same scans were used to generate both AI- and HI-based virtual implant plans. Pre- and postoperative scans were registered to allow accurate comparison between placed and planned implants. Three plans were evaluated per case: clinically placed implant (HI-place), HI-planned implant (HI-plan), and AI-planned implant (AI). Evaluations included angular deviation relative to adjacent teeth and prosthetic wax-ups, vertical and horizontal bone thickness, proximity to vital structures, implant dimensions, planning time, and planning repeatability.

**Results:**

The Friedman test indicated no significant differences in implant location among the 3 approaches (*P* > .05). Angular deviations were similar across methods for both the neighboring tooth (HI-place = 7.01 ± 4.7°; HI-plan = 7.30 ± 5.6°; AI = 6.86 ± 3.7°) and the wax-up (HI-place = 5.22 ± 3.0°; HI-plan = 4.55 ± 3.0°; AI = 5.50 ± 4.9°). Implant diameter and length selections also did not differ significantly across HI-place (4.06 ± 0.4 mm; 10.61 ± 1.4 mm), HI-plan (4.06 ± 0.4 mm; 10.45 ± 1.4 mm), and AI (4.05 ± 0.5 mm; 10.39 ± 1.4 mm). AI was significantly faster (*P* < .05), requiring 150 s vs 429 s for HI-plan, and showed excellent repeatability with zero deviation between repeated plans.

**Conclusions:**

AI-driven virtual implant planning achieved clinically acceptable placement comparable to expert clinicians in single-implant rehabilitation, with superior efficiency and repeatability, supporting its use in routine presurgical workflows.

**Advances in knowledge:**

Integrating AI for implant location and dimensions selection, with accuracy comparable to expert clinicians, can streamline the digital implant workflow, reduce planning time, and allow clinicians to focus on verifying the AI-generated plan prior to clinical transfer, thereby increasing the time available for the actual treatment procedure.

## Introduction

Achieving predictable outcomes in dental implant rehabilitation requires meticulous preoperative planning to fully assess anatomical constraints and ensure customized implant positioning.^1^ Three-dimensional (3D) imaging technologies, particularly cone-beam CT (CBCT) and intraoral scanning (IOS), have considerably boosted clinical decision-making by providing detailed insights into patient-specific anatomical conditions, including the quantity and quality of remaining bone and its spatial relationship to adjacent teeth, vital anatomical structures, and the anticipated prosthetic outcome represented by the designed wax-up, all with superior precision and accuracy.^2–6^

Furthermore, recent advancements in digital technologies capable of processing 3D imaging data have significantly improved the precision and clinical applicability of dental implant planning.^5^^,^^7^ These innovations enable the accurate transfer of virtual planning into the clinical environment through (in-house) 3D printing technologies, facilitating the fabrication of surgical guides for static guided implant placement with enhanced accuracy and predictability.^8^^,^^9^

The digital transformation in oral healthcare increasingly demands that clinicians be thoroughly trained and possess the necessary knowledge to effectively utilize these tools. Adoption of novel digital technologies into everyday practice remains a complex and often lengthy process.^10–12^ Moreover, implant planning is inherently influenced by human variability, as treatment decisions based on human intelligence (HI) differ according to the clinician’s expertise, clinical experience, and individual decision-making approach. These differences can introduce bias and undermine the uniformity and repeatability of treatment results.^10^^,^^13^^,^^14^

As a result, artificial intelligence (AI) has been incorporated to optimize key stages of the digital implant workflow. AI systems have been validated for various preoperative planning tasks, such as segmenting the teeth, jaws, maxillary sinus, mandibular canal, prosthetic crowns, and implant restorations, as well as registering CBCT and IOS datasets in a wide range of clinical applications.^15–28^ Ultimately, AI enables the creation of individualized dental implant plans by utilizing detailed virtual patient models derived from the segmentation and registration of CBCT and IOS data, allowing precise adaptation to each patient’s unique anatomical features.^29–33^ Previous studies have demonstrated that AI can perform these tasks with expert-level accuracy while achieving significantly greater efficiency and higher consistency compared to traditional digital planning.^10^^,^^33^

Existing literature on AI in virtual implant planning has mainly examined implant location prediction or compared AI-generated plans with human planning, yet it has not validated AI performance against actual clinical placements. Therefore, it is unclear whether AI-based implant plans achieve clinically acceptable accuracy with respect to key anatomical and prosthetic structures. Thus, this study aimed to address this uncertainty by providing clinical evidence and evaluating the degree of correspondence between AI-driven, human expert, and actual clinical implant planning in single-tooth rehabilitation. The analysis evaluates agreement in implant location selection, orientation, and spatial relationships to adjacent teeth, vital anatomical structures, and the prosthetic wax-up. Accordingly, the aim of this research was to assess the performance of an AI-based platform in dental implant planning by comparing it with expert planning and actual clinical scenarios, with a focus on anatomical accuracy, prosthetic alignment, implant dimension selection, planning efficiency, and repeatability.

## Methods

Approval for this retrospective study was granted by the Ethics Committee of University Hospitals Leuven (UZ Leuven, Leuven, Belgium; S66447), and all procedures were performed in full compliance with ICH-GCP standards, the Declaration of Helsinki, and the Oviedo Convention.^34^ All data were anonymized, and the requirement for informed consent was waived.

### Dataset

A dataset comprising 32 patients (16 males and 16 females; mean age = 45 ± 13 years) who received single-tooth implants at the University Hospitals Leuven (UZ Leuven, Belgium) was analyzed. For each patient, preoperative and postoperative CBCT scans, as well as an IOS, were available. Imaging acquisition involved CBCT scans obtained via the NewTom VGI Evo platform (Cefla) and IOS data collected using the TRIOS 3 IOS system (3Shape).

### Inclusion and exclusion criteria

Inclusion criteria consisted of a solitary missing anterior or posterior mandibular tooth or solitary missing anterior or premolar maxillary tooth, along with the availability of a postoperative CBCT scan obtained within 6 months following the preoperative scan, performed at the request of the treating experts for clinical follow-up of the placed implants (eg, when in proximity to vital structures such as the mandibular canal, incisive nerve, or nasal floor) or for the other pathological conditions unrelated to the implant sites (eg, cysts or tumors). Exclusion criteria comprised cases presenting with missing posterior maxillary teeth (to eliminate potential confounding effects from sinus-grafting or sinus-elevation procedures), cases requiring soft-tissue or bone augmentation, and cases with low-quality or motion-affected CBCT imaging. The 32 cases included 20 missing posterior teeth (14 mandibular molars and 6 premolars), whereas 12 concerned missing anterior maxillary teeth.

### Dataset preparation

CBCT datasets (ie, preoperative and postoperative scans) were exported in Digital Imaging and Communications in Medicine (DICOM) format, whereas all IOS acquisitions were saved as Standard Tessellation Language (STL) files. Registration of each preoperative CBCT volume to its corresponding postoperative dataset was performed using a voxel-based approach implemented in the Amira software package (Thermo Fisher Scientific). The initial registration was carried out by an expert in prosthetic dentistry (E.A.D.), after which a specialist in maxillofacial radiology (R.C.F.) independently reviewed and confirmed spatial accuracy. Upon verification, the registered preoperative CBCT datasets were re-exported in DICOM format for subsequent analytical processing.

Following image registration, the pre- and postoperative CBCT datasets were imported into a cloud-based AI platform designed for virtual patient construction (Relu Creator). Through automated AI-based segmentation, the software generated detailed 3D models encompassing the dentition, alveolar structures, inferior alveolar nerve canal, maxillary sinus, and the expert-placed implant for postoperative CBCT scans. To enhance the preoperative model, IOS data were incorporated and automatically registered to the corresponding CBCT dataset using the platform’s proprietary AI-driven registration algorithm.

This multimodal integration enabled the simultaneous visualization of hard and soft-tissue structures, producing a realistic digital representation of the clinical situation and enhancing preoperative planning by clarifying the spatial relationship between tissues to determine the optimal implant location. The AI-based segmentation and registration tools implemented in the Relu Creator platform have been individually validated through prior technical and preclinical studies.^15^^,^^18^^,^^20^^,^^24^^,^^35–40^

### Implant planning approaches

The study involved a comparative analysis of 3 implant planning methodologies applied to each individual case:

1. Expert clinical placement (HI-place): Clinically placed implants were inserted by specialists in maxillofacial surgery and periodontology, guided by preoperative CBCT scans. All decisions regarding implant specifications (ie, location and dimensions) were determined entirely prior to the study, based on the experts’ clinical judgment.
2. Expert virtual planning (HI-plan): For each case, an implant prosthodontist (B.E.) manually planned the implant using the planning tools available within the Relu platform. The planning process was guided by a compromise between available bone volume, prosthetic requirements (as determined by the designed wax-up), and aesthetic considerations. All virtual implant plans were subsequently reviewed and approved by an expert in implant dentistry (P.L.).
3. AI-driven virtual implant planning (AI): Within the Relu platform, the operator can access the implant planning module by selecting the tooth number for which a virtual implant is to be placed. After selection of the tooth number and implant brand, the AI-based system automatically proposes the implant location and selects the implant dimensions, leveraging prior training data to balance predefined clinical parameters with the AI-generated prosthetic wax-up.^29^^,^^30^^,^^41^ The predetermined planning criteria were as follows:
  - A minimum distance of ≥2 mm from vital anatomical structures, including the mandibular canal for mandibular molars and premolars, the incisive canal or nasal floor for maxillary anterior teeth, and the maxillary sinus floor for posterior maxillary premolars.
  - A minimum of ≥1.5 mm from adjacent natural teeth.
  - A minimum of ≥3 mm from neighboring implants.

**Figure 1:**
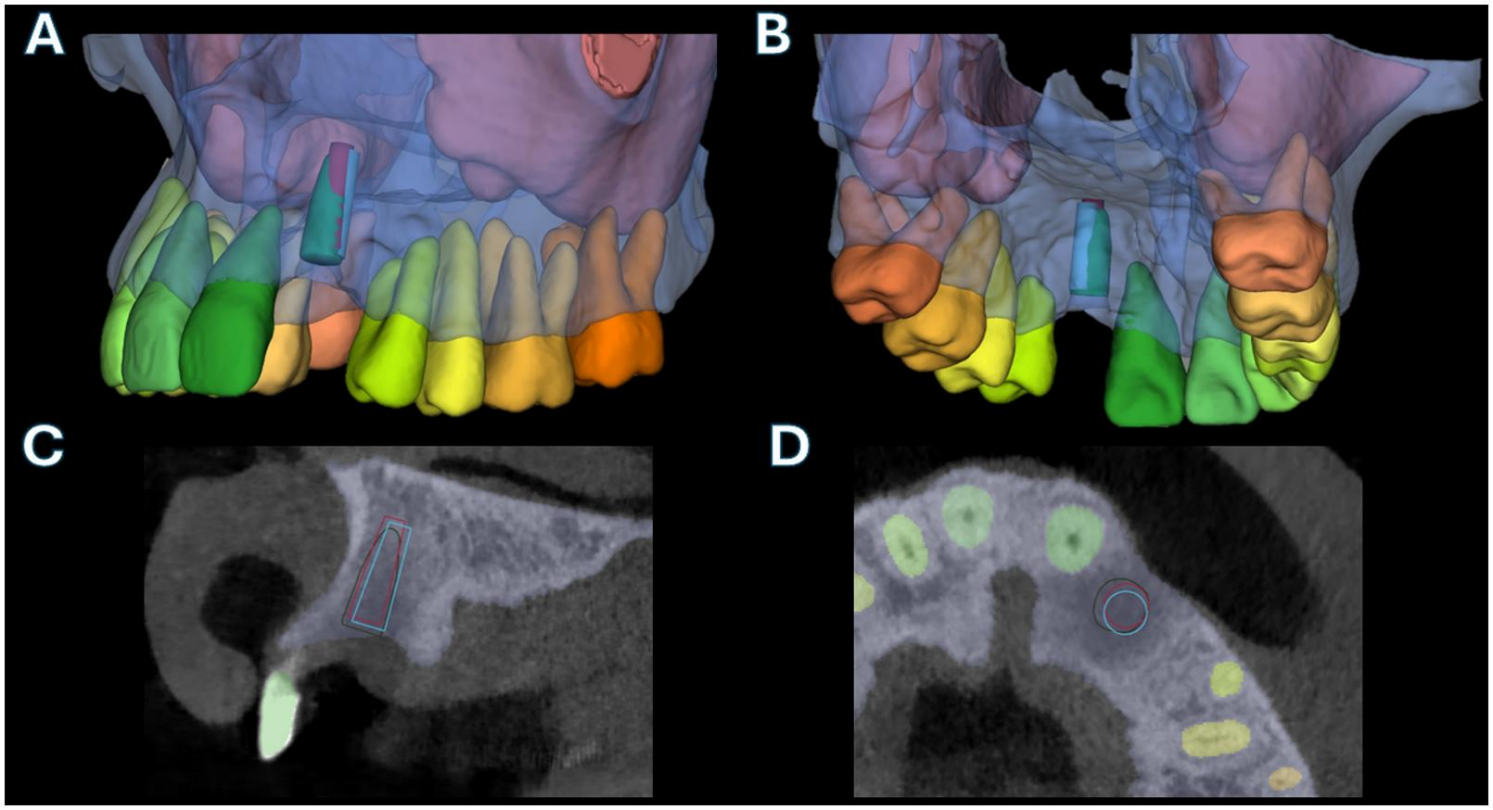
Comparison of the expert-placed implant (green), expert-planned implant (blue), and AI-planned implant (red) in a case involving a maxillary left lateral incisor. The 3 implant positions are shown relative to key anatomical structures, including the maxillary complex, adjacent dentition, and the maxillary sinus, from the labial (A) and palatal (B) perspectives, while the relationship among the 3 implants is shown in the sagittal (C) and axial (D) views.

For each case, a comparison was conducted among 3 3D implant models: the clinically placed implant and 2 implant planning approaches, namely, AI-based and human expert-based. Accordingly, the 3 implant models were exported as STL files: the clinically placed implant (HI-place), the expert-planned implant (HI-plan), and the AI-generated implant (AI). The clinically placed implant was automatically segmented from the postoperative CBCT dataset using the same AI-driven platform, which had been previously trained for high-accuracy implant segmentation. This approach ensured that residual imaging artifacts and blooming effects did not significantly influence the comparisons between the planned and placed implants. The segmentation accuracy of this platform has been validated in prior peer-reviewed studies (Figure 1).^36^^,^^38^

### AI performance in selecting implant location and dimensions

The STL files corresponding to the 3 implant models (HI-place, HI-plan, and AI), as well as the AI-segmented maxilla/mandible and dentition, were imported into 3-matic software (Materialise) for quantitative analysis. This evaluation assessed the spatial relationships between the implants and surrounding anatomical structures or wax-ups, allowing for a comprehensive comparison across the 3 implant planning approaches.

#### Relation to adjacent anatomical structures

**Figure 2:**
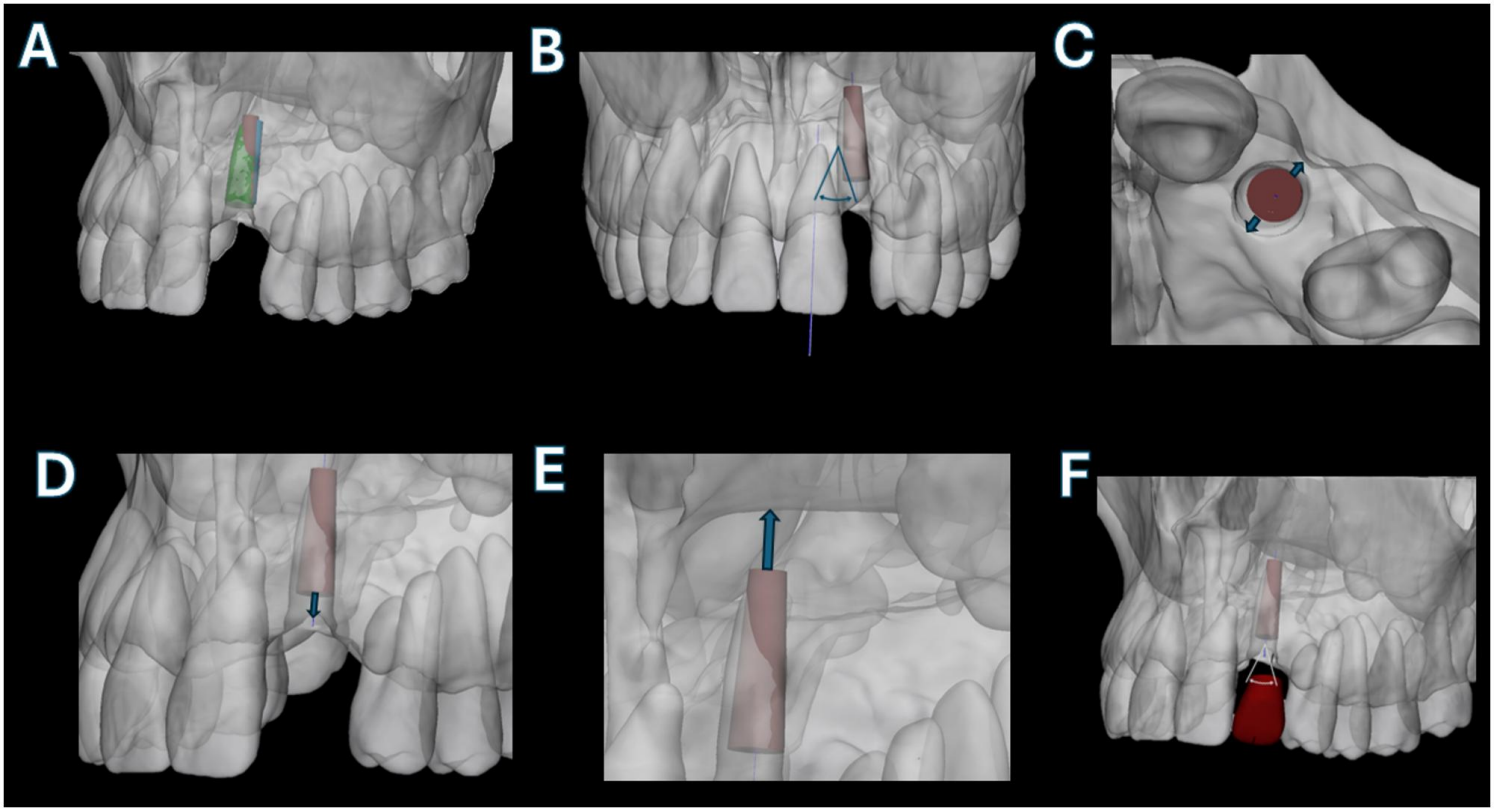
Anatomical and prosthetic assessment of planned and clinically placed implants. (A) Three implants for a missing maxillary left lateral incisor shown within the 3D-segmented model of the maxillary complex: expert-placed (green), expert-planned (blue), and AI-planned (red). (B) Angular deviation between the implant’s long axis and that of the adjacent anterior tooth. (C) Labial and palatal bone thickness surrounding the implant. (D) Crestal bone height measured from the implant platform to the alveolar crest. (E) Distance from the implant apex to the nasal floor. (F) Angular deviation between the implant’s long axis and that of the expert-designed wax-up. Abbreviations: AI = artificial intelligence–planned implants; HI-place = human intelligence-placed implants; HI-plan = human intelligence–planned implants.

Figure 2 illustrates the parameters used to evaluate implant positioning. The 3 implant planning approaches (HI-place, HI-plan, and AI) were assessed according to the following anatomical criteria:

1. Angular deviation relative to the adjacent tooth: Measured as the angle (in degrees) between the long axis of the implant and that of the neighboring tooth.
2. Crestal bone level at the implant platform: Defined as the vertical distance (in millimeters) from the center of the implant platform to the most coronal point of the surrounding crestal bone.
3. Buccal/labial and lingual/palatal bone thickness: Determined as the horizontal thickness of bone (in millimeters) at both the buccal/labial and lingual/palatal aspects of the implant platform.
4. Proximity of the implant apex to critical anatomical structures: Measured as the vertical distance (in millimeters) from the apex of the implant to the closest vital structure, including the mandibular canal, incisive canal, nasal floor, or maxillary sinus floor, depending on implant location.

#### Relationship with the prosthetically designed wax-up

The angular deviation (in degrees) between the long axis of the planned or placed implant and the long axis of the prosthetically driven wax-up, as defined by an expert in prosthetic dentistry (E.A.D.).

#### Implant diameter and length selection

For each missing tooth, the implant diameter and length (in millimeters) were compared across the HI-place, HI-plan, and AI-selected implants. This analysis was designed to quantify differences in implant dimensions selected by the AI-based system relative to those chosen by human operators during both clinical placement and expert planning.

### Time consumption

Planning efficiency was assessed by recording the duration (in seconds) needed to complete implant planning for both the AI-assisted workflow and the expert manual method (HI-plan). Total planning time was defined as the elapsed period from the initial upload of imaging data to the export of the finalized implant model, and measurements were obtained using a digital stopwatch.

### Repeatability

To assess intra-method repeatability, each implant planning workflow (AI and HI-plan) was done twice, generating a first and second set of plans for comparison. The difference between the repeated plans was assessed using median surface deviation (MSD) and root mean square deviation (RMS). The MSD reflects the average variation across the implant surface, while the RMS provides an overall measure of spatial discrepancy. A value of zero indicates identical implant positioning between repetitions.

### Statistical analysis

All statistical analyses were performed using MedCalc Statistical Software (MedCalc Software Ltd.). The sample size was calculated based on the study of Elgarba et al.,^41^ using *α* = .05, power = 0.80, a medium effect size (Cohen’s *d* = 0.5), a mean implant deviation of 0.1 mm, and an SD of 0.13 mm between AI- and HI-driven planning, resulting in a required total of 32 participants to detect a statistically significant difference. Data normality was assessed using the Shapiro-Wilk test. Continuous variables were summarized as median ± interquartile range for non-normally distributed data and as mean ± SD for normally distributed data. Differences among planning methods relative to anatomical landmarks and wax-up were assessed using the Friedman test with Wilcoxon signed-rank post hoc comparisons. Implant dimension variations were analyzed via repeated-measures analysis of variance (ANOVA) with Tukey’s correction, while paired *t*-tests compared time consumption and intra-method repeatability between AI and HI workflows. Statistical significance was defined as *P* < .05.

## Results

### Anatomical acceptability of AI-Planned implants

No statistically significant differences were observed among the 3 approaches (HI-place, HI-plan, and AI) for any of the anatomy-related metrics (*P* > .05), as determined by the Friedman test. The angular deviation between the implant’s long axis and that of the adjacent anterior tooth was comparable across all groups: HI-place = 7.01 ± 4.7°, HI-plan = 7.30 ± 5.6°, and AI = 6.86 ± 3.7° (*P* = .94). These findings demonstrate that the AI planning tool achieved implant positioning closely aligned with that of experienced human operators. Table 1 provides a detailed comparative analysis of implant location selection by HI-place, HI-plan, and AI approaches relative to adjacent anatomical landmarks.

**Table 1:** Assessment criteria for human intelligence (HI)-place/plan and artificial intelligence (AI)-planned implants, evaluated using reference anatomical landmarks for single-tooth implant rehabilitation cases in anterior and posterior regions, as well as across the full dataset.

Metric	HI-place	HI-plan		AI	HI-place	HI-plan	AI	HI-place	HI-plan	AI	*P*-value (entire dataset comparison)
	Posterior (*n* = 20)				Anterior (*n* = 12)			Entire dataset (*n* = 32)			
	Median ± IQR										
**Angle with anterior adjacent tooth (°)**	7.25 ± 3.2	6.73 ± 6.8	6.15 ± 4.3		6.30 ± 8.7	8.65 ± 5.0	7.6 ± 2.4	7.01 ± 4.7	7.30 ± 5.6	6.86 ± 3.7	.94
**Crestal bone height (mm)**	0.83 ± 0.7	1.10 ± 0.5	1.11 ± 0.6		1.10 ± 1.8	1.90 ± 1.7	1.55 ± 1.3	0.98 ± 1.2	1.24 ± 0.7	1.17 ± 0.8	.05
**Buccal/labial bone thickness (mm)**	3.05 ± 2.3	2.41 ± 2.8	2.12 ± 2.9		1.80 ± 0.8	1.80 ± 0.7	1.50 ± 0.7	2.09 ± 1.9	2.00 ± 1.6	1.67 ± 2.1	.90
**Lingual/palatal bone thickness (mm)**	1.68 ± 1.3	1.46 ± 1.4	1.54 ± 1.3		1.30 ± 0.6	1.20 ± 0.9	1.00 ± 0.7	1.48 ± 1.0	1.36 ± 1.1	1.44 ± 1.1	.33
**Distance to vital structure (mm)**	5.23 ± 2.4	6.01 ± 2.8	6.26 ± 2.0		4.75 ± 4.18	5.10 ± 2.0	5.70 ± 2.0	5.23 ± 3.4	5.29 ± 2.6	6.11 ± 2.1	.16

The Friedman test revealed no statistically significant differences (*P* > .05) among the implant planning approaches and clinical implant placement across the entire dataset. Abbreviations: ° = degree; AI = artificial intelligence–planned implants; HI-place = human intelligence-placed implants; HI-plan = human intelligence–planned implants; IOR = interquartile range; mm = millimeter.

### Prosthetic acceptability of AI-planned implants

In relation to the angular deviation between the long axis of the placed or planned implants and the long axis of the prosthetically designed wax-up, no statistically significant difference was observed between the AI-planned implants and those planned or placed by the human experts (*P* = .06), as indicated by the Friedman test. The AI-planned implants demonstrated an angular deviation of 5.50° ± 4.9°, compared with 5.22° ± 3.0° for the expert-placed implants and 4.55° ± 3.0° for the expert-planned implants.

### Implant dimension selection

The comparison of implant diameter and length selection among the HI-place, HI-plan, and AI approaches revealed no statistically significant differences, as shown by repeated-measures ANOVA (*P* > .05). No significant differences were observed for implant diameter (*P* = .96) or implant length (*P* = .27), indicating a high level of agreement among the 3 methods.

**Figure 3:**
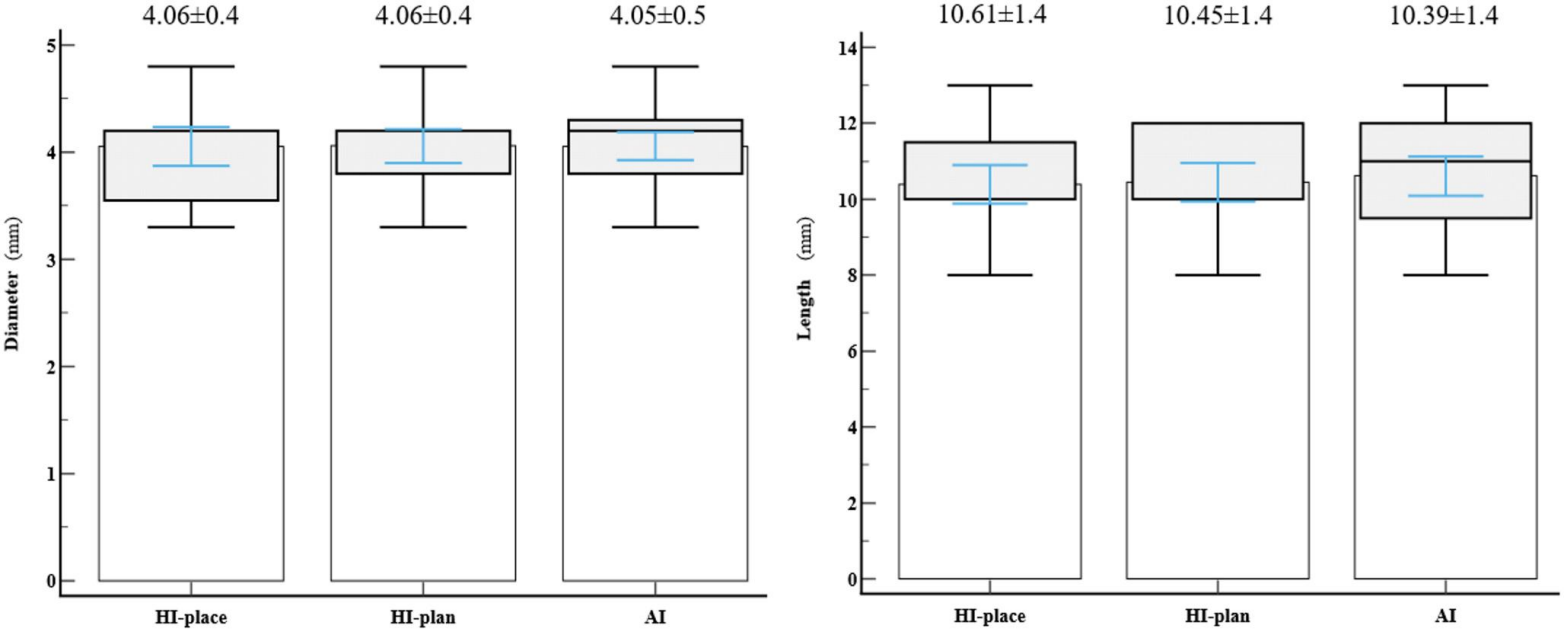
Comparison of implant diameter and length selection between human intelligence (HI)-placed/planned and artificial intelligence (AI)-planned implants.

The mean implant diameter and length selected by the AI approach were 4.05 ± 0.5 mm and 10.39 ± 1.4 mm, respectively. This closely matched the selections for HI-place (4.06 ± 0.4 mm, 10.61 ± 1.4 mm) and HI-plan (4.06 ± 0.4 mm, 10.45 ± 1.4 mm). Figure 3 illustrates the comparison of implant diameter and length selection among the 3 approaches.

### Time consumption

Implant planning time differed significantly between the AI-assisted and expert-based (HI-plan) workflows (*P* < .001). Remarkably, the AI approach achieved a nearly 3-fold increase in efficiency, requiring a mean duration of 150 ± 7.3 s, whereas the HI-plan workflow took 429 ± 113 s on average. These results highlight the substantial time-saving potential of AI-driven implant planning compared with conventional expert-based methods.

### Repeatability

A statistically significant difference (*P* < .05) was observed between the AI and HI-plan approaches for both MSD and RMS values. In contrast to HI-plan, which produced MSD and RMS deviations of 0.47 ± 0.23 mm and 0.79 ± 0.32 mm, respectively, AI yielded perfect consistency, showing no measurable surface deviation (0.00 ± 0.00 mm for both metrics).

## Discussion

Although AI has revolutionized the digital implant workflow by automating numerous steps, the clinical validation of emerging AI-based implant planning solutions remains limited in the current literature.^10^^,^^33^ A key strength of this study is its thorough evaluation of an AI-driven implant planning system, benchmarked to both expert-generated virtual plans and surgically expert-placed implants. These dual-level human expert references provide a robust and clinically relevant assessment of AI performance. Results demonstrate that the AI system achieved implant location selection for single-tooth replacement comparable to that of surgical experts while maintaining a high level of repeatability and offering substantially increased efficiency.

The anatomical validity of the AI, HI-place, and HI-plan approaches was assessed by examining their spatial relationships to adjacent anatomical structures, which served as consistent reference landmarks, allowing for reliable comparisons across the different approaches.^42^^,^^43^ The results demonstrated that AI-generated plans, human-generated plans, and expert-placed implants consistently aligned the implant along the long axis of neighboring teeth while maintaining favorable horizontal and vertical bone relationships and remaining within a safe distance from vital anatomical structures. No significant differences were observed among the 3 approaches, highlighting that AI can achieve precision comparable to expert clinicians while ensuring anatomical safety and suitability.

Additionally, successful dental implant planning depends on harmonizing the anatomical constraints of the alveolar bone with the desired prosthetic outcomes, a process often informed by a preoperative diagnostic wax-up.^44^ In the present study, AI accounted for both anatomical constraints and prosthetic design, resulting in clinically comparable peri-implant bone dimensions and alignment with the wax-up. Specifically, AI-planned implants demonstrated a buccal/labial bone thickness of 1.67 ± 2.1 mm, lingual/palatal thickness of 1.44 ± 1.1 mm, and crestal bone height of 1.17 ± 0.8 mm. Comparable values were observed in the HI-place and HI-plan approaches: HI-place showed 2.09 ± 1.9 mm buccal/labial, 1.48 ± 1.0 mm lingual/palatal, and 0.98 ± 1.2 mm crestal bone thickness, while HI-plan measured 2.00 ± 1.6 mm buccal/labial, 1.36 ± 1.1 mm lingual/palatal, and 1.24 ± 0.7 mm crestal height. Regarding implant-wax-up angulation, AI demonstrated an angular deviation of 5.50 ± 4.9°, compared with 5.22 ± 3.0° for HI-place and 4.55 ± 3.0° for HI-plan, indicating that AI achieves clinically comparable prosthetic alignment. The results of this study reveal that AI can achieve prosthetically driven implant location selection while maintaining sufficient bone support, emphasizing its clinical reliability.

The selection of implant dimensions (ie, diameter and length) by the AI tool showed a high level of concordance with both HI-place and HI-plan approaches. The AI-selected implants had mean dimensions of 4.05 ± 0.5 mm in diameter and 10.39 ± 1.4 mm in length, closely matching those selected by HI-place (4.06 ± 0.4, 10.61 ± 1.4 mm) and HI-plan (4.06 ± 0.4, 10.45 ± 1.4 mm). No statistically significant differences were observed among the 3 approaches, indicating that AI is capable of reliably selecting implant dimensions comparable to expert decision-making. These findings are consistent with previous reports demonstrating that AI-driven planning can closely replicate expert-level judgments in implant selection and configuration.^29^^,^^30^

A significant benefit of using AI in implant planning is its superior time efficiency and repeatability. In the present study, the AI tool performed the planning process almost 3 times faster than human operators, while maintaining excellent repeatability across multiple plans. In contrast, expert-based planning (HI-plan) showed lower repeatability, evidenced by greater MSD and RMS values. These findings show AI’s credibility as a reliable and effective tool that can offer consistent outcomes regardless of human variability, such as tiredness or emotional state. This observation is consistent with previous research that supports the incorporation of AI into digital dentistry workflows, including anatomical segmentation, IOS-CBCT registration, and preoperative planning.^17^^,^^19^^,^^20^^,^^37^^,^^38^^,^^45^^,^^46^

To address the current gap in clinical evidence comparing AI-based dental workflows in implant planning, this study provides a clinical assessment of AI-driven virtual implant planning for single-tooth rehabilitation. The performance of the AI tool was uniquely evaluated against both routine expert clinical planning and actual implant placement, thereby situating the assessment within a real-world clinical workflow. However, the study has certain limitations: it relied on a single data source, and the included cases involved only single implant planning in the mandible and anterior maxilla, without addressing the posterior maxilla. To further enhance the generalizability of these findings, additional studies are warranted that incorporate multiple data sources, include cases with missing posteriors in the maxilla or multiple missing teeth, and compare AI planning outcomes across different anatomical regions (anterior, posterior, maxillary, and mandibular). Additionally, prospective investigations comparing AI-generated plans with both expert planning and actual clinical outcomes will be essential to fully validate the tool across diverse clinical situations.

**Figure 4:**
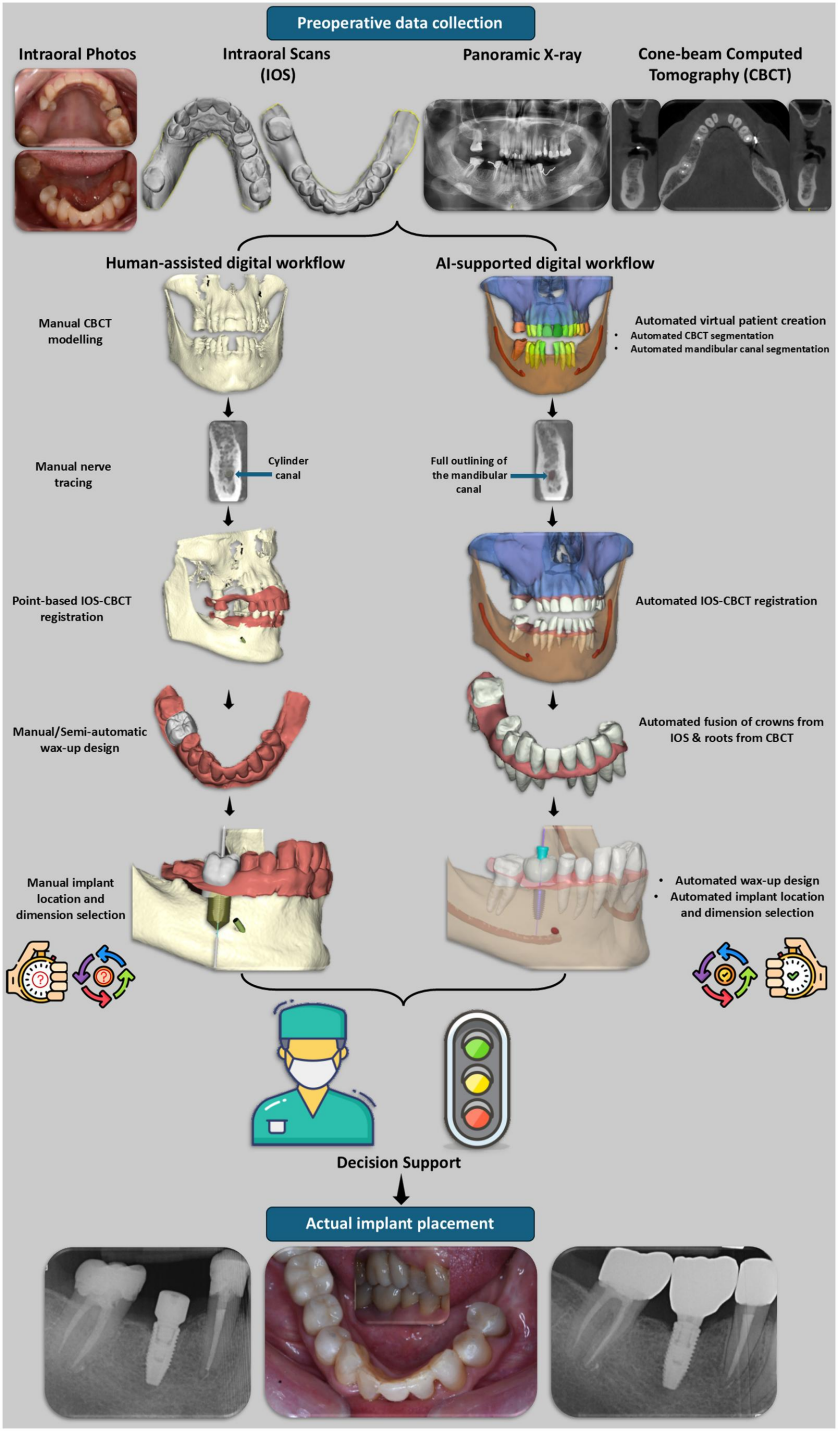
Comparison of human-assisted and artificial intelligence (AI)-supported digital dental implant workflows. Both approaches follow the same fundamental steps, beginning with anatomical landmarks segmentation and intraoral scan to cone-beam CT (CBCT) registration, followed by wax-up design and virtual implant placement. However, the AI-supported workflow executes these steps with markedly greater speed and repeatability compared with the human-assisted approach.

Furthermore, although the segmentation of the clinically placed implant from postoperative CBCT scans was performed using a trained AI model with high segmentation accuracy to minimize imaging artifacts and blooming effects, the influence of blooming artifacts cannot be entirely eliminated and may still affect the accuracy of measurements when compared with the planned implants. Moreover, it is important to emphasize that the integration of AI into the digital implant workflow is not intended to replace human expertise. A final verification must always be performed by a qualified clinician with fundamental knowledge of implant dentistry. Rather, AI serves as an assistant tool to provide a fast, consistent, and reproducible planning approach. This can significantly enhance clinical efficiency by allowing clinicians to devote more time to patient care while only requiring a few seconds to review, approve, or adjust an AI-generated plan, rather than spending substantial time planning each case from scratch, a process inherently subject to human variability. Figure 4 provides an overview of the benefits of incorporating AI into the routine digital implant planning workflow.

## Conclusion

The AI tool evaluated for implant location and dimensions selection demonstrated outcomes that were anatomically and prosthetically comparable to expert planning and clinical placement of single implants in the posterior mandible and the upper jaw anterior to the maxillary sinus. Notably, the AI system exhibited approximately a 3-fold improvement in time efficiency and improved repeatability compared with human expert planning. These findings highlight the potential of integrating AI into virtual implant workflows as an assistive planning tool, representing a valuable enhancement to digital presurgical planning. Nonetheless, further research involving larger and more diverse datasets, as well as multi-implant scenarios, is warranted to validate a broader clinical applicability with long-term reliability and increased levels of complexity.
